# Congenital Perineal Groove: A Rare Benign Neonatal Anomaly Mimicking Perineal Trauma

**DOI:** 10.7759/cureus.96462

**Published:** 2025-11-10

**Authors:** Rajarajan Paulpandian

**Affiliations:** 1 Pediatrics/Neonatology, Chettinad Hospital and Research Institute, Chennai, IND

**Keywords:** anogenital malformation, benign, congenital perineal groove, neonatal, perineal malformation

## Abstract

Congenital perineal groove is a rare, often under-recognized anomaly of the anogenital region, characterized by a non-keratinized mucosal sulcus extending from the posterior vaginal fourchette to the anterior margin of the anus. We report a term female neonate weighing 2.9 kg, born via normal vaginal delivery to a primigravida mother after an uneventful perinatal period, in whom a mucosal groove was noted extending from the posterior vaginal fourchette to the anal verge during routine examination. A perineal tear was initially suspected; however, the uncomplicated delivery argued against this diagnosis. A literature review confirmed the finding as a congenital perineal groove - a benign developmental anomaly. Recognition of this condition at birth is crucial to avoid misdiagnosis as perineal trauma or ambiguous genitalia. Conservative management with meticulous local hygiene and regular follow-up remains the mainstay of treatment; surgical correction is reserved for persistent or complicated cases. Awareness of this rare yet benign entity among clinicians ensures accurate diagnosis, appropriate counseling, and optimal management.

## Introduction

Congenital perineal groove is a rare malformation of the anogenital region, characterized by a linear, moist, non-keratinized mucosal sulcus extending from the posterior vaginal fourchette to the anterior aspect of the anus. Although the congenital perineal groove is recognized as a rare condition, the exact incidence remains unknown, as most available data are limited to isolated case reports and small case series [1]. Initially believed to occur exclusively in females, a few male cases have since been reported, suggesting a shared embryological defect in external genital development [2]. The anomaly likely arises from incomplete fusion of the perineal raphe during external genital development. Normally, the labioscrotal folds and ectoderm fuse in the midline to form the perineal raphe, separating it from the anal folds. A defect in this process results in a mucosal-lined sulcus between the vaginal fourchette and anus - the defining feature of a perineal groove. Proposed mechanisms include persistence of the urorectal septum, failure of median genital fold fusion, or a vestigial open cloacal duct. Overall, it represents a localized developmental anomaly arising during the complex division and fusion of cloacal and perineal structures in early embryogenesis [2]. Stephens first described the condition with three features: (1) a moist groove between the vaginal fourchette and anus, (2) normal vestibular formation (urethra and vagina), and (3) hypertrophy of the minoral tails extending posteriorly to the anus or encircling it [3]. The non-epithelialized region typically undergoes spontaneous epithelialization by two years of age, reaffirming its benign nature [4]. In a small proportion of cases, local infection or inflammation may occur due to exposure of the mucosal surface to moisture or secretions, occasionally leading to urinary tract infection [5]. Early recognition by clinicians prevents unnecessary investigations, facilitates appropriate counseling, and alleviates parental anxiety.

## Case presentation

**Figure 1:**
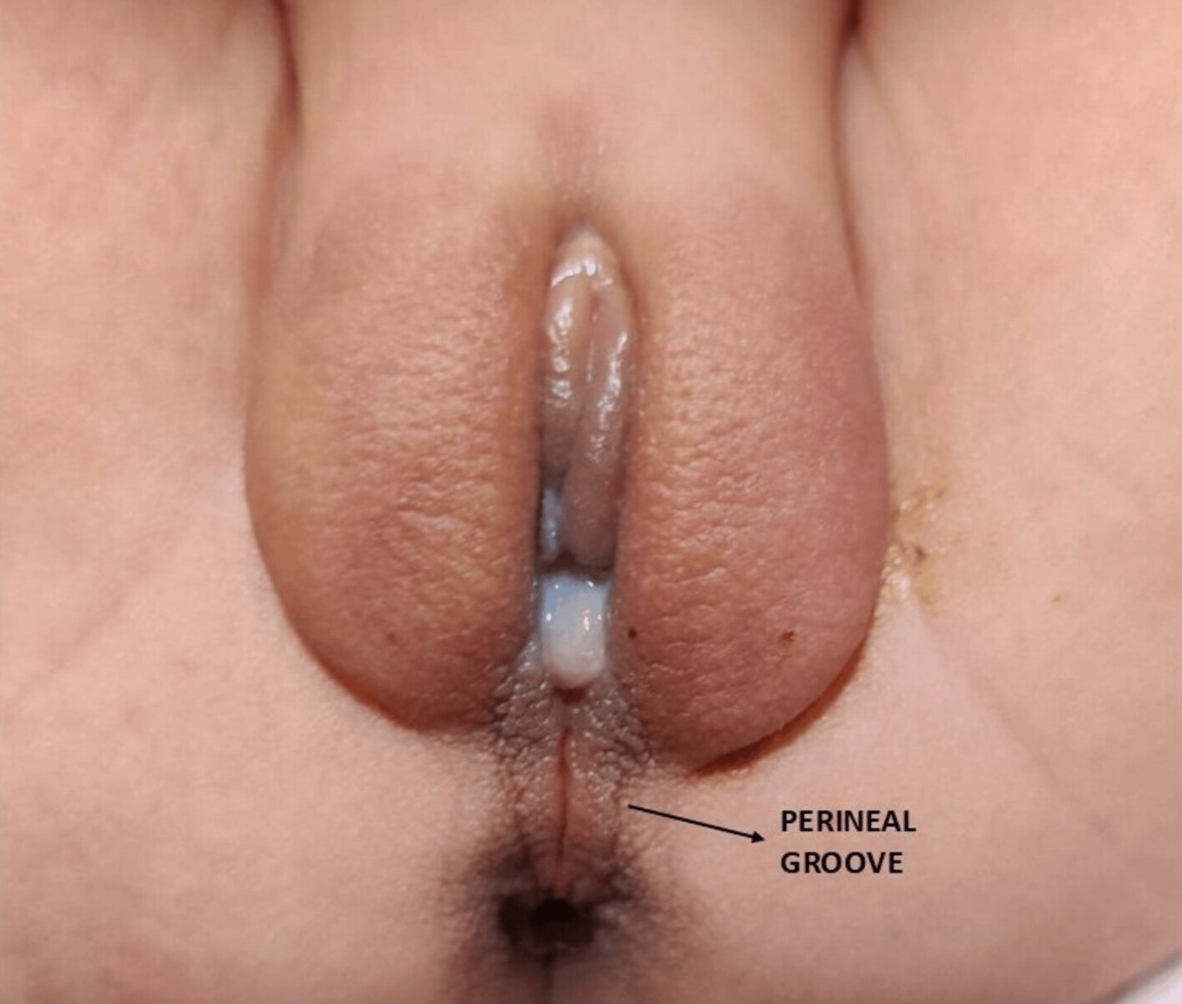
Wet, erythematous sulcus connecting the vaginal and anal openings, suggestive of a perineal groove.

A term female neonate weighing 2.9 kg was delivered vaginally to a 25-year-old primigravida after an uneventful antenatal and intrapartum period. The mother had no history of diabetes, infection, or drug exposure during pregnancy, and there was no consanguinity. The neonate cried immediately after birth, with an Apgar score of 8 and 9. On routine examination, a linear, moist, non-bleeding mucosal groove was observed extending from the posterior vaginal fourchette to the anterior margin of the anal verge (Figure 1).

The surrounding perineal skin appeared healthy, with no erythema, edema, or ulceration. Both urethral and anal openings were normally positioned and patent, with no evidence of fistulous communication, discharge, or inflammation. The external genitalia were otherwise normal for a female neonate. No history of perineal trauma or obstetric injury was present. A perineal tear or ulceration was initially considered; however, the intact perineal tissues, absence of bleeding, and uneventful delivery made these unlikely. Systemic examination revealed no associated anomalies, and the infant passed urine and meconium normally. Based on the characteristic appearance and exclusion of other differentials such as perineal tear, fistula, and perianal pyramidal protrusion, a diagnosis of congenital perineal groove was made. The parents were counseled regarding the benign, self-resolving nature of the condition. Conservative management with meticulous local hygiene, gentle cleansing with sterile water, and avoidance of irritants was advised. Regular follow-up was recommended to monitor for spontaneous epithelialization. Progressive epithelialization was noted, with partial healing observed at the six-month follow-up.

## Discussion

The findings in our case of congenital perineal groove align with descriptions reported in existing literature concerning its appearance, location, and natural course. Additionally, this report broadens understanding by emphasizing the differential diagnosis of perineal lesions across different age groups, which is crucial for accurate recognition and avoidance of misdiagnosis. Congenital perineal groove is an uncommon developmental anomaly of the anogenital region, most frequently seen in female neonates [1]. It typically presents as a linear, moist, erythematous, non-keratinized mucosal sulcus extending from the posterior vaginal fourchette to the anterior margin of the anus. The surrounding perineal skin remains intact and healthy, and both urethral and anal openings are normally positioned and patent. The condition is often identified incidentally during a routine newborn examination, as affected infants are typically asymptomatic. Typically, the perineal groove occurs as an isolated anomaly and is not associated with other congenital malformations [6], although rare cases have been reported in males and in those with associated genitourinary abnormalities. Because of its atypical appearance, a perineal groove can easily be mistaken for other pathological conditions such as perineal tears, contact dermatitis, anal fissures, ulcerated hemangioma, lichen sclerosus, perianal pyramidal protrusion, or even signs suggestive of sexual abuse. Such misinterpretation can lead to parental anxiety and unnecessary investigations or interventions. A thorough history, absence of trauma, and characteristic lesion morphology usually suffice for diagnosis. Table 1 summarizes the distinguishing features of the perineal groove from other perineal lesions.

**Table 1:** Differential diagnosis of CPG according to age of presentation.

Age group	Differential diagnosis	Key distinguishing features of the congenital perineal groove (CPG)
Neonatal (at birth)	Perineal tear/birth trauma [7]	History of complicated delivery, irregular wound edges, ecchymosis or bleeding; absent smooth mucosal sulcus.
	Perianal pyramidal protrusion [8]	Small, soft, protrusion in the perineal median raphe anterior to the anus; CPG is a depressed linear groove extending between the fourchette and the anus.
	Anorectal malformation/perineal fistula [9]	Absent or abnormally placed anal opening, failure to pass meconium, or associated fistula; CPG shows normal anal position and patent orifice.
Early infancy (≤6 months)	Diaper/irritant contact dermatitis [10]	Diffuse erythema and scaling involving convex surfaces; CPG is sharply demarcated, midline, and persistent.
	Anal fissure [11]	Linear tear in the keratinized anoderm of the posterior midline, whereas CPG is a midline non-keratinized mucosal extension from the posterior fourchette to the anterior anal verge
Later infancy to childhood (>6 months to 12 years)	Lichen sclerosus [12]	Chronic, porcelain-white plaques or atrophic patches, often pruritic; not a linear wet mucosal groove.
	Ulcerated hemangioma/vascular lesion [13]	Raised erythematous mass with ulceration; Doppler is diagnostic. CPG is flat, non-vascular, and stable since birth.
	Suspected sexual abuse (injury) [14]	Irregular lacerations or ecchymoses inconsistent with birth history; CPG is symmetric, midline, congenital, and non-traumatic.

The natural course is usually benign, with gradual epithelialization occurring spontaneously within the first two years of life [4]. Conservative management, including meticulous local hygiene, avoidance of irritants, and regular follow-up, remains the standard of care. Topical antibiotics or emollients may be applied if mild mucous discharge is present. Surgical excision is rarely indicated and reserved for cosmetic reasons when spontaneous resolution does not occur or in cases complicated by recurrent infection [15]. Parental education forms a key component of management. Informing caregivers about the benign, self-limiting nature of the condition helps alleviate anxiety and ensures adherence to follow-up until complete epithelialization is achieved.

## Conclusions

Congenital perineal groove is a rare but benign developmental anomaly. Early recognition and clear parental counseling are essential to prevent misdiagnosis and ensure optimal outcomes. Conservative management with observation remains the treatment of choice unless complications develop. By documenting this case, we aim to increase clinical awareness of this rare entity, highlight its benign nature, and emphasize the importance of differentiating it from pathological perineal lesions. 
